# A GIS software-based method to identify public health data belonging to address-defined communities

**DOI:** 10.1093/jamia/ocae235

**Published:** 2024-08-26

**Authors:** Amanda M Lam, Mariana C Singletary, Theresa Cullen

**Affiliations:** Epidemiology Division, Pima County Health Department, Tucson, AZ 85714, United States; Epidemiology Division, Pima County Health Department, Tucson, AZ 85714, United States; Pima County Health Department, Tucson, AZ 85714, United States

**Keywords:** geographic information systems, public health surveillance, geocoding, health equity, American Indian and Alaska Native

## Abstract

**Objective:**

This communication presents the results of defining a tribal health jurisdiction by a combination of tribal affiliation (TA) and case address.

**Materials and Methods:**

Through a county-tribal partnership, Geographic Information System (GIS) software and custom code were used to extract tribal data from county data by identifying reservation addresses in county extracts of COVID-19 case records from December 30, 2019, to December 31, 2022 (*n* = 374 653) and COVID-19 vaccination records from December 1, 2020, to April 18, 2023 (*n* = 2 355 058).

**Results:**

The tool identified 1.91 times as many case records and 3.76 times as many vaccination records as filtering by TA alone.

**Discussion and Conclusion:**

This method of identifying communities by patient address, in combination with TA and enrollment, can help tribal health jurisdictions attain equitable access to public health data, when done in partnership with a data sharing agreement. This methodology has potential applications for other populations underrepresented in public health and clinical research.

## Background and significance

Disaggregating public health data is a critical part of identifying health inequities. During the COVID-19 pandemic, 38% of United States states reported COVID-19 case data by race and ethnicity, allowing racial disparities experienced by impacted communities to be potentially mitigated by actions such as targeted vaccine outreach.^1^ For tribal nations, as governments independent of the United States, disaggregation is also integral to tribal sovereignty. In Arizona, public health surveillance data includes a tribal affiliation (TA) field to facilitate the transfer of tribal data to their respective tribal health jurisdiction. However, in practice, TA is frequently missing from surveillance records, such that tribal data can default to the county jurisdiction. Furthermore, American Indian/Alaska Native (AI/AN) individuals are underrepresented in surveillance data when they are misclassified as white or another race, though data linkage can help correct racial misclassification.^2^^,^^3^

These gaps in tribal surveillance data availability and racial misclassification obscured the full extent of COVID-19’s disproportionate impact on AI/AN populations. In April 2020, data showed that AI/AN people, who made up 4.6% of the state’s population, accounted for 16% of COVID-19 deaths.^4^ Although Arizona was one of the few states that reported the number of confirmed COVID-19 cases and deaths for AI/AN populations early in the pandemic and published the data by zip code with tribal approval,^5–7^ tribal entities could not always obtain a complete picture of the state of their own communities, hindering tribal decision-making during the public health crisis. The pandemic highlighted how the existing public health surveillance system could not truly support Indigenous data sovereignty—the rights of Indigenous Peoples to govern and access data about their communities, lands, and resources, regardless of where the data is stored.^8^ It also showed the need for public health applications of the CARE principles, a set of guidelines for data governance of tribal data.^9^^,^^10^

Given limitations of relying solely on TA, this article explores the idea of identifying tribal data by address for a community that shares zip codes with the county. Geographic Information System (GIS) software can be run within an intranet without passing any protected health information to an outside third-party, ensuring Health Insurance Portability and Accountability Act (HIPAA)-compliance.^11^ Prior to geocoding, addresses can be corrected and standardized; this pre-processing has been shown to improve geocoding rate and positional accuracy.^12^

## Objective

This communication presents the results of defining a tribal health jurisdiction by a combination of TA and address. The article introduces the idea of “address-defined communities.” An address-defined community is a community defined by a set of address points, such that members do not have to be within the same pre-defined area, like zip code, census tract, or county, or share a demographic, or other filterable database field typically used for public health analysis. Delineating the reservation as an address-defined community allows for the retrieval of tribal data not marked by a TA, and helps locate records that cannot be filtered by zip code.

## Methods

**Figure 1.**
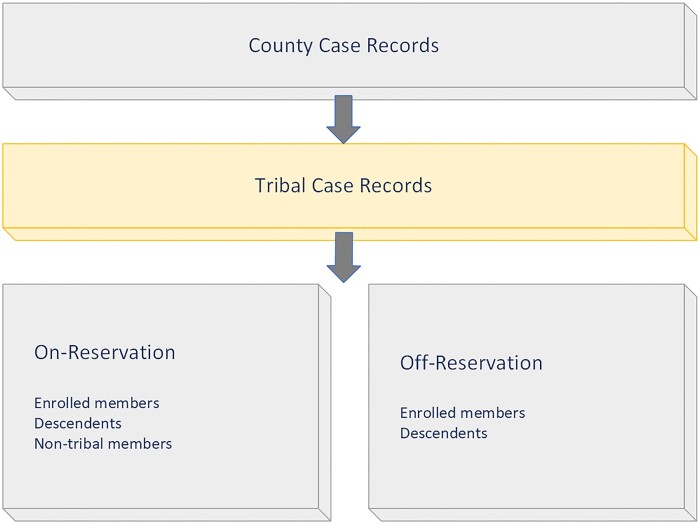
Types of tribal records that can be found in a county extract.

The local health department established a formal partnership with the tribal health department by signing an intergovernmental data sharing agreement in early 2023. The source data is extracted by the Arizona Department of Health Services for the Pima County jurisdiction from 2 state surveillance databases: Medical Electronic Disease Surveillance Intelligence System (MEDSIS) and Arizona State Immunization Information System (ASIIS). A case record in MEDSIS refers to a confirmed or probable infectious disease surveillance case based on a laboratory test result, and a vaccination record refers to the administration of a single dose of a vaccine recorded in ASIIS. The MEDSIS dataset consisted of COVID-19 case records from December 30, 2019, to December 31, 2022 (*n* = 374 653), and the ASIIS dataset consisted of COVID-19 vaccination records from December 1, 2020, to April 18, 2023 (*n* = 2 355 058). Cases include Pima County and tribal residents, as well as non-residents who were seen at a health facility located in Pima County (Figure 1). Reservation addresses were received from the tribal development office on March 15, 2023. The geocoder references addresses from a spatial dataset created by the county GIS department. The county dataset is updated nightly and was last accessed by the data tool in August 2023 to generate the final results. The geocoder uses a minimum match score of 85 and accepts address information in 2 fields: street address and zip code.

**Figure 2.**
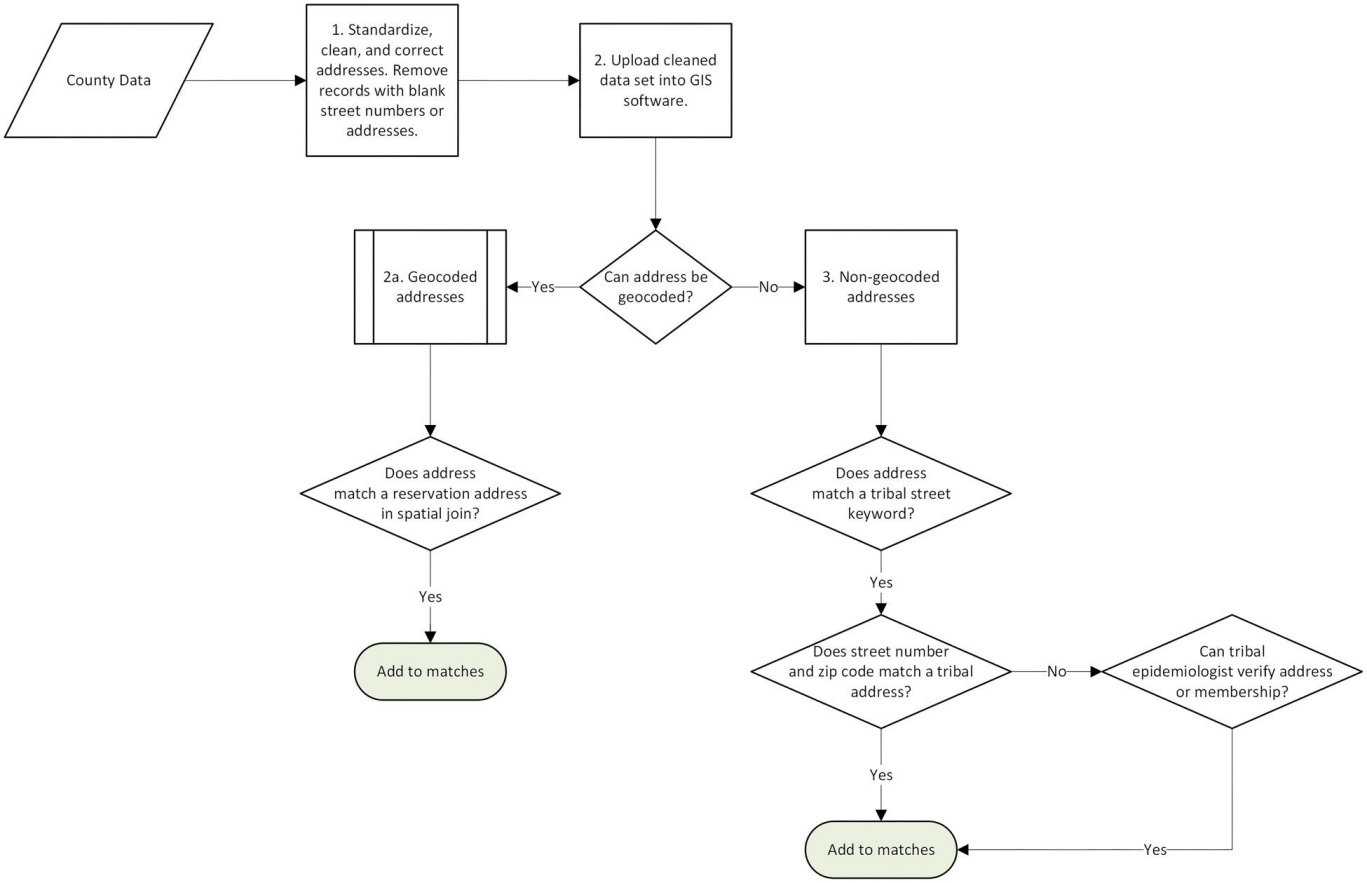
Flowchart of algorithm to identify records with a reservation address.

The datasets were first processed with custom R code, which standardized and corrected the addresses using regular expressions that adjust spelling and formatting to match what the geocoder is expecting (Figure 2). Blanks, PO Boxes, and numberless addresses were also removed. The cleaned datasets were then geocoded in GIS software and a spatial join identified addresses that were within 150 feet of a tribal address, the distance at which variations in where a point is located for the same address are captured without including a non-tribal address. The few non-tribal points that were less than 150 feet from a tribal address were manually excluded. The set of records that could not be geocoded were further processed by R code that identified addresses that matched a keyword, defined as a unique part of the full tribal street name or a unique word that commonly appears in tribal addresses. The flagged records were included as a match if the street number and zip code matched a known tribal address, or if the tribal epidemiologist found the person in their database. The address standardization code was created by reviewing the tribal addresses that could not be geocoded and formulating regular expressions to capture spelling or formatting variations. Each regular expression was tested against a list of Pima County addresses to ensure that similar Pima County street names would not be included, and all matches that did not contain a zip code associated with the reservation were manually reviewed.

For data analysis, the R program references the following database fields: Patient Home Street Address, Patient Home Zip Code, and TA from MEDSIS, and Patient Mailing Address, Patient Zip Code, and Reservation from ASIIS. At the beginning of the pandemic, MEDSIS only allowed 1 race to be selected and later allowed for multiple races to be selected. The program filters by all available race fields for single and multiple-race selections of AI/AN in MEDSIS and the 2 available single-race fields in ASIIS.

## Results

Of the 374 653 COVID-19 case records, 2801 reservation matches were found (Table 1). Of these, 48% of the records did not have TA associated with the record. 68% of the records were marked with a race of AI/AN. Of the 2886 records with a TA, half were for people who lived on-reservation and half were for people who lived off-reservation or who had a PO Box, did not have an address on file, or had a non-geocodable address. In total, 4233 COVID-19 case records from the county extract were found to belong to the tribal health jurisdiction.

**Table 1.** Analysis of matches by reservation address and tribal affiliation for COVID-19 case and vaccination records.

	COVID-19 case records (December 30, 2019-December 31, 2022)	COVID-19 vaccination records (December 1, 2020-April 18, 2023)
Total # of county records	374 653	2 355 058
Total # of records on-reservation	2801	2993
With tribal affiliation^a^	1464 (52.27%)	797 (26.6%)
W/o tribal affiliation	1337 (47.74%)	2196 (73.37%)
With AI/AN race	1900 (68%)	1347 (45.00%)
Total # of records w/tribal affiliation	2886	797^b^
On-reservation	1464 (50.73%)	797 (100%)
Off-reservation or unknown residence	1422 (49.27%)	0 (0%)
With AI/AN race	2661 (92.20%)	341 (42.8%)
Total # of tribal records identified	4223	2993
With AI/AN race	3167 (74.99%)	1347 (45.00%)
With tribal affiliation	2886 (68.34%)	797 (26.6%)

a The reported percentage of on-reservation records with a tribal affiliation does not represent the actual percentage of tribally affiliated people who live on-reservation.

b The vaccination database only has a Reservation field; Tribal Affiliation field is not available.

Abbreviation: AI/AN, American Indian and Alaska Native.

Of the 2 355 058 COVID-19 vaccination records, 2993 reservation matches were found. Of these, nearly three-quarters of the records did not have a TA. 45% of matches had a race of AI/AN. Of the 797 vaccination records with a TA, all were for on-reservation patients with 43% containing AI/AN race. In total, 2993 vaccination records from the county extract were found to belong to the tribal health jurisdiction.

Among all reservation case and vaccination matches, GIS software identified 94% and 96% of the records. Address processing raised the geocoding rate to 95% and uncovered an additional 172 case records and 118 vaccination records (Table 2). An examination of non-geocoded addresses that ended up being matches found the following patterns: variations in spelling, abbreviations, and formatting; historical changes in zip code and street name; typos in street address, street number, and zip code. About 1% of matches were found to have a non-reservation zip code, which on manual review were found to be typos in the zip code with an otherwise correct reservation address.

**Table 2.** Evaluation of geocoding and address processing.

	COVID-19 cases (*n* = 374 653)	COVID-19 vaccinations (*n* = 2 355 058)
Geocoding rate w/o address processing	89.66%	92.91%
Geocoding rate of subset w/address processing	95% (*n* = 354 406)	95% (*n* = 2 284 254)
# of matches w/o address processing	2629	2875
# of matches w/address processing	2801	2993
# of additional matches w/address processing	172 (6.14%)	118 (3.94%)
% of matches with non-reservation zip code	1.3% (37/2801)	0.80% (24/2993)

## Discussion

The methodology identified 1.91 times as many COVID-19 case records and 3.76 times as many vaccination records for cases living on the reservation as filtering by TA. The percentage of matches without TA (48%-73%) represents the “lost” data that the tribe did not have access to about their jurisdiction during the pandemic. When filtering by TA alone, only half had a reservation address. Address alone can identify someone who lives on a reservation and can include tribal members, descendants, and non-members. From a public health perspective, there is utility in identifying all case records on a reservation regardless of membership, because everyone living within the reservation boundary is under the same tribal public health authority.

**Figure 3.**
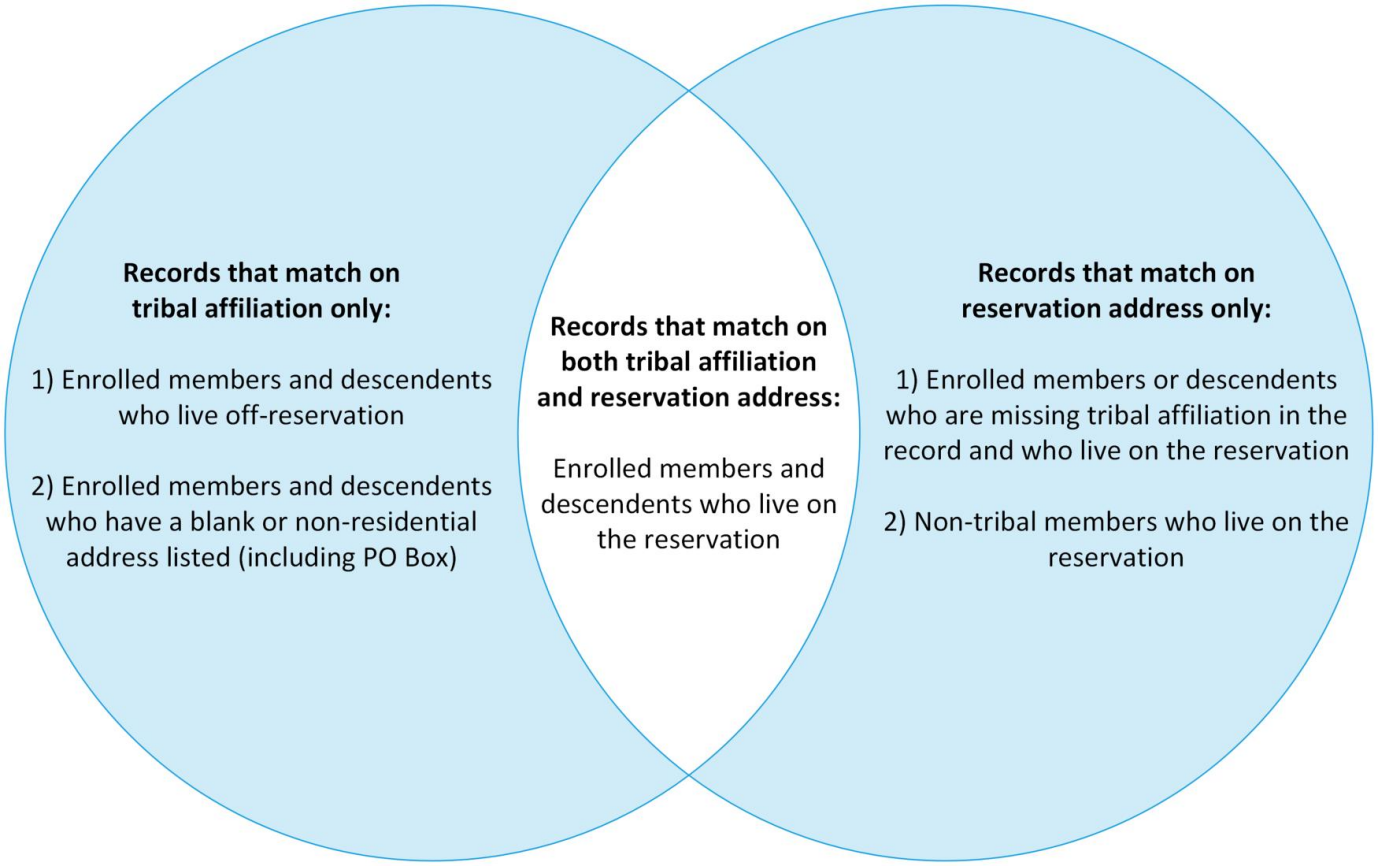
Summary of people identified using combination of address and tribal affiliation fields.

A combination of address and TA will generate the largest, most relevant, and most flexible data set for tribal nations, particularly if the reservation shares their zip codes with non-tribal lands. Being able to filter by both address and TA rather than just by TA enables tribal nations to identify (1) all records on the reservation regardless of TA (including tribal records that would’ve been missing due to TA not being collected with the data), and (2) records of tribal members or descendents who live off-reservation (by subtracting the subset of records that are on-reservation) (Figure 3). This approach should only be used in the context of joint decision-making within a partnership, ideally guided by a data sharing agreement.

The difference in TA percentages between case and vaccination data (52% versus 27%) may be due to differences in database design, data entry interface, as well as workflow differences among the diversity of healthcare providers and case investigators that may be submitting COVID-19 data. Vaccination data is also skewed because the same patient often appears multiple times for each vaccination in the series that they obtain. Although GIS software alone identified a majority of case and vaccination matches, the additional matches found from the non-geocoded addresses can be especially significant for smaller populations or rare conditions. Address processing also accounted for cultural differences in naming conventions to improve geocoding rates.

### Future developments

Improvements to the address processing code and development of similar code for other tribal nations could use natural language processing to build address standardization and correction models specific to tribal addresses, and to improve the ability to sort through the non-geocoded addresses for matches. For tribal nations with addresses that are not already part of an existing geocoder, the reference data used by the geocoder can be expanded using GIS software. Linkage software could be used to verify tribal enrollment across public health data systems and tribal registries. The availability of various tools allows for flexibility in how a tribal nation defines their health jurisdiction.

The county is currently in the process of automating the tool to generate regular extracts more sustainably for our tribal partner for different state datasets. A gold standard tribal extract would contain all cases for people who reside at a tribal address along with records containing non-tribal addresses that meet other desired criteria such as TA and tribal provider. In practice, the challenge of cleaning and standardizing free text addresses, of sifting through the non-geocodable addresses for a match, whether with code or manually, and of not having control over the data collection, limits a tool’s ability to meet the gold standard. In automating the process, the manual portions will need to be codified or removed without significantly compromising the quality of the extract.

The notion of address-defined communities has applications for other minority groups. For example, Pacific Islanders are often excluded from research because they are a small and geographically dispersed population, but this method could help create an aggregated dataset so that community members and researchers can generate important population health knowledge. Communities that span political borders can also be defined by address and their associated data compiled for further analysis.

### Limitations

The following people can be missed if certain fields are incomplete in their record: enrolled member or descendent who lives on the reservation + no TA or reservation address, non-tribal person who lives on the reservation + no reservation address, member or descendent who lives off-reservation (including unhoused) + no TA, a tribal member with more than one tribal identity because only one TA can be entered. False or missed matches can occur if the GIS software geocodes an address incorrectly or if an incorrect address is listed in the record, though these errors are mitigated by validation using the mapping software and custom code.

## Conclusion

This article describes a preliminary evaluation of a novel methodology for identifying public health data belonging to a community based on patient address. By using GIS software in combination with an address processing program, a greater number of matches were found than with existing database filters. This method is a potential tool for supporting tribal health data access needs and for advancing data equity in public health and clinical research for underrepresented populations.

## Data Availability

The data underlying this article cannot be shared publicly due to the data being protected health information.
